# Pre-diagnosis oophorectomy, estrogen therapy and mortality in a cohort of women diagnosed with breast cancer

**DOI:** 10.1186/bcr3560

**Published:** 2013-10-24

**Authors:** Hazel B Nichols, Amy Trentham-Dietz, Polly A Newcomb, Kathleen M Egan, Linda J Titus, John M Hampton, Kala Visvanathan

**Affiliations:** 1 Epidemiology Branch, National Institute of Environmental Health Sciences, 111 T.W. Alexander Drive, Research Triangle Park, Durham, NC 27709, USA; 2 Department of Population Health Sciences, University of Wisconsin, 610 Walnut St., Madison, WI 53726, USA; 3 University of Wisconsin Paul P. Carbone Comprehensive Cancer Center, 610 Walnut St., Madison, WI 53726, USA; 4 Fred Hutchinson Cancer Research Center, 1100 Fairview Ave. N., PO Box 19024, Seattle, WA 98109, USA; 5 H. Lee Moffitt Cancer Center & Research Institute, 12902 Magnolia Drive, Tampa, FL 33612, USA; 6 Dartmouth Medical School, 1 Medical Center Drive, Lebanon, NH 03756, USA; 7 Sidney Kimmel Comprehensive Cancer Center at Johns Hopkins, 401 N. Broadway, Baltimore, MD 21231, USA

## Abstract

**Introduction:**

Pre-diagnosis oophorectomy and estrogen therapy could impact mortality due to breast cancer and cardiovascular disease (CVD) among breast cancer survivors. Elective bilateral oophorectomy at the time of hysterectomy for benign conditions is not uncommon among US women.

**Methods:**

We examined the association between pre-diagnosis total abdominal hysterectomy with bilateral salpingo-oophorectomy (TAHBSO) and both overall and cause-specific mortality in the Collaborative Breast Cancer Studies cohort. Medical history and prior estrogen use were collected during standardized telephone interviews. Vital status, including date and cause of death, was obtained by linkage with the National Death Index. Multivariate hazard ratios (HR) and 95% confidence intervals (CI) for cause-specific mortality were calculated using Cox proportional hazards regression.

**Results:**

Seventeen percent (N = 1,778) of breast cancer survivors (mean age at diagnosis = 63.5) reported pre-diagnosis TAHBSO. During follow-up (mean = 9.5 years), 2,856 deaths occurred, including 1,060 breast cancer deaths and 459 CVD deaths. Breast cancer deaths occurred a median of 5.1 years after diagnosis; CVD deaths occurred further from diagnosis (median = 9.7 years). Women who reported pre-diagnosis TAHBSO had a 16% decrease in all-cause mortality (HR = 0.84; 95% CI: 0.76, 0.92) compared to those with an intact uterus and ovaries. This overall decrease reflected a 27% lower breast cancer mortality among women who never used postmenopausal hormones (HR = 0.73; CI: 0.55, 0.96) and 43% lower CVD risk among women who reported using estrogen (HR = 0.57; CI: 0.39, 0.83) after TAHBSO.

**Conclusions:**

Information on prior TAHBSO and estrogen use can inform risk of death from both breast cancer and cardiovascular disease among breast cancer survivors.

## Introduction

With a growing population of >2.7 million breast cancer survivors in the United States [1], identifying factors that contribute to long-term mortality is of increasing importance. Pre-diagnosis behaviors can impact survival from breast cancer and medical conditions, such as cardiovascular disease [2-5]. For example, higher levels of physical activity and lower body mass index (BMI) prior to breast cancer diagnosis are associated with improved all-cause and cause-specific survival [3,6-8]. A beneficial effect of pre-diagnosis bilateral oophorectomy on breast cancer-specific mortality has been reported in some [9], but not all [10], studies of women in the general population and among patient populations of women at high risk of developing breast cancer due to *BRCA*1/2 mutation status [11]. However, all-cause mortality among women with breast cancer could be adversely affected by increases in deaths due to heart disease, stroke, or fracture that may be associated with ovarian removal [10,12-15]. These opposing risks and benefits may be further influenced by the use of unopposed estrogen therapy after surgery [2,10,12,14,16-18].

In the United States, total abdominal hysterectomy with bilateral salpingo-oophorectomy (TAHBSO) is often performed for benign conditions such as uterine fibroids; an estimated 15% of US women have their uterus and ovaries removed by age 60 [19,20]. Identifying prognostic factors at breast cancer diagnosis for both cancer and non-cancer outcomes can inform risk of death among breast cancer survivors and guide appropriate chronic disease prevention strategies. To examine the impact of pre-diagnosis TAHBSO and estrogen therapy on cause-specific mortality, we analyzed data from a cohort of 10,443 US women diagnosed with postmenopausal invasive breast cancer.

## Methods

This analysis was performed with data from the Collaborative Breast Cancer Study Cohort [6,21,22]. Cohort members participated as cases in one of four consecutive population-based case-control studies of invasive breast cancer. Participant identification, enrollment, and data collection procedures were maintained across studies. All participants provided verbal informed consent at the time of the phone-based survey. Standard protocols for each study were approved by Institutional Review Boards at the University of Wisconsin-Madison, Harvard School of Public Health, and Dartmouth Medical School.

### Study population

Study participants were female residents of Wisconsin, Massachusetts, or New Hampshire with an incident invasive breast cancer reported to each state’s cancer registry during the study enrollment years. Eligibility was limited to women with listed telephone numbers, driver’s licenses (if younger than 65 years), and registry-reported dates of diagnosis. Women ages 50 to 79 were eligible in the first study (1992 to 1995); and ages 20 to 69 in subsequent studies (1997 to 2001; 2001 to 2004; 2004 to 2007). During 1992 to 2001 participants were enrolled in Wisconsin, Massachusetts, and New Hampshire; in 2001 to 2007 women were enrolled in Wisconsin only. Across the four studies, 21,713 eligible participants were identified. Physicians refused contact with 359 (1.7%), 743 (3.4%) were deceased, 624 (2.9%) could not be located and 2,794 (12.9%) refused to participate. Therefore, 17,193 (79.2%) eligible breast cancer survivors were interviewed. Thirty-eight interviews were considered unreliable by the interviewers, leaving 17,155 interviews available for analysis.

### Exposure assessment

In each study, participants completed a 40-minute structured telephone interview that elicited detailed information on lifestyle and demographic factors, postmenopausal hormone use, and personal and family medical history. Study participants reported whether they had surgery to remove the uterus or ovaries, the type of surgery (hysterectomy and/or oophorectomy, including number of ovaries removed), and age at surgery. Participants were asked a standard history of postmenopausal hormone use including formulation, route of administration, age started, frequency of each episode of use, total duration, and time since last use.

In three of the four pooled studies (1992 to 1995; 1997 to 2001; 2001 to 2004), menopause was defined as the absence of menses for six consecutive months not attributable to surgery, chemotherapy, radiation, or other reasons. In the most recent study (2004 to 2007), menopause was defined as 12 consecutive months without menses. In all studies, women who reported bilateral oophorectomy prior to breast cancer diagnosis were categorized as postmenopausal. BMI was calculated as weight (kg)/tallest adult height (m)^2^ during the one- to five-year period prior to breast cancer diagnosis.

### Outcome assessment

Vital status information, including date and underlying cause of death, was collected through December 31, 2009 through linkage with the National Death Index. Cause of death was categorized according to the International Classification of Disease (ICD-10) code as breast cancer-specific mortality (C50), cardiovascular (CVD) mortality (I00 to I09, I11, I13, I20 to 51), stroke mortality (I60 to I69), or all-cause mortality [23].

### Population for analysis

For consistency in ages at enrollment across studies, this pooled analysis was limited to women age 50 and older at breast cancer diagnosis (N = 13,253). We further excluded women who were premenopausal at diagnosis (N = 1,352) or had unknown menopausal status (N = 620), or who had a previous history of cancer (except non-melanoma skin cancer) (N = 614). Ninety-three records from women with discordant ages at bilateral oophorectomy and hysterectomy and 41 records with missing ages for both procedures were excluded. To exclude gynecologic surgeries performed as part of breast cancer treatment, 84 women who reported bilateral oophorectomy or hysterectomy at the same age or after breast cancer diagnosis were excluded. Six records were excluded based on partial interview completion. After these exclusions were applied, 10,443 women were eligible for analysis. Among the eligible breast cancer survivors, 1,778 (17.0%) reported having a prior TAHBSO and 6,913 (66.2%) reported having an intact uterus and ovaries at breast cancer diagnosis; therefore, records from 8,691 breast cancer survivors contributed to this analysis. The remaining 1,752 records included 1,641 women who reported hysterectomy alone or unilateral oophorectomy with or without hysterectomy, and 111 women with missing information on previous gynecologic surgery. On average, women were interviewed 1.3 years (standard deviation (SD) = 0.5; range: 0.2 to 4.9) after breast cancer diagnosis.

### Statistical analysis

All mortality comparisons were made relative to women who reported having an intact uterus and ovaries and never using postmenopausal hormones at breast cancer diagnosis. Kaplan Meier curves were produced to display mortality curves; formal statistical comparison of the difference in slopes was conducted with log rank tests [24]. For survival analyses, the time scale was defined as time since breast cancer diagnosis (years) and person-time was accrued from date of interview. Participants either developed the event of interest (breast cancer mortality/cardiovascular disease mortality/all-cause mortality) or were administratively censored on December 31, 2009. In statistical models of mortality within five years of diagnosis, person-time was accrued from date of interview to the event of interest or administrative censoring at five years after the diagnosis date. For analyses of five-year survivors, person-time was accrued from five years after diagnosis until the event of interest or administrative censoring on December 31, 2009.

Cox proportional hazards regression was used to calculate multivariate hazard ratios (HR) for all-cause mortality. The Fine and Gray method was used to fit proportional cumulative incidence-associated subhazards regression models for breast cancer and CVD-specific mortality to account for non-breast and non-CVD disease deaths as competing events [25,26]. *A priori,* final estimates were adjusted for stage of diagnosis, state, study enrollment years, age at diagnosis, BMI one to five years before breast cancer diagnosis, smoking status at breast cancer diagnosis, and family history of breast cancer as potential confounders. *P* values ≤0.05 were considered to be statistically significant. All statistical analyses were performed with Stata 12 software (StataCorp, College Station, TX, USA).

### Sensitivity analyses

Across the four studies, the majority of participants were enrolled in Wisconsin (69%) and were diagnosed with local invasive disease (64%). We conducted sensitivity analyses to evaluate whether inclusion of participants in Massachusetts and New Hampshire or those with regional or distant disease influenced our results.

## Results

A total of 2,856 deaths occurred during 82,201 person-years (mean = 9.5 years; SD = 4.8) contributed by 8,691 women. The greatest proportion (37.1%) of deaths were due to breast cancer (N = 1,060), followed by coronary heart disease (16.1%, N = 459) and stroke (5.4%, N = 153).

Study participant characteristics according to gynecologic surgery status are presented in Table 1. Overall, the exposure groups had similar distributions for age at breast cancer diagnosis, state of residence, and parity. TAHBSO was more frequently reported in earlier study years. Women who had undergone TAHBSO were less likely to smoke, had lower education, and were more likely to be overweight or obese and have a first-degree family history of breast cancer than women who reported having an intact uterus and ovaries. Use of estrogen-only postmenopausal hormone formulations was largely restricted to women who underwent TAHBSO compared to women with an intact uterus and ovaries (estrogen-only formulations are not recommended for women with an intact uterus due to the increased risk of endometrial cancer). Women who reported TAHBSO were slightly more likely to be diagnosed with local stage breast cancer relative to women with an intact uterus and ovaries (Table 1).

**Table 1:** Participant characteristics according to gynecologic surgery history prior to breast cancer diagnosis, Collaborative Breast Cancer Studies

	**TAHBSO**^**a**^**(N = 1,778)**		**Intact uterus and ovaries (N = 6,913)**		***P*-value**^**b**^
Age at diagnosis, mean (SD)	63.5	6.9	63.2	6.8	0.08
Study enrollment years, %					
1992-1995	876	49.3	3,177	46.0	0.01
1997-2001	527	29.6	2,071	30.0	
2001-2004	172	9.7	690	10.0	
2004-2007	203	11.4	975	14.1	
State, %					
WI	1,205	67.8	4,787	69.2	0.3
MA	433	24.4	1,568	22.7	
NH	140	7.9	558	8.1	
Smoking status at diagnosis, %					
Never	955	53.7	3,237	46.8	<0.001
Former	564	31.7	2,376	34.4	
Current	256	14.4	1,258	18.2	
Missing	3	0.2	42	0.6	
Education, %					
High school diploma or less	1,070	60.2	3,924	56.8	0.04
Some college	383	21.5	1,538	22.2	
College degree or higher	320	18.0	1,424	20.6	
Parity, mean (SD)^c^	3.1	1.6	3.1	1.6	0.5
Body mass index, %					
Underweight (< 18.5 kg/m2)	24	1.3	102	1.5	0.002
Normal (18.5-24.9 kg/m2)	641	36.1	2,813	40.7	
Overweight (25-29.9 kg/m2)	626	35.2	2,276	32.9	
Obese (≥ 30 kg/m2)	450	25.3	1,542	22.3	
Missing	37	2.1	180	2.6	
Family history of breast cancer, %					
Yes	418	23.5	1,445	20.9	0.04
Missing	27	1.5	127	1.8	
Postmenopausal hormone therapy, %					
None	539	30.3	4,541	65.7	<0.001
Estrogen only	1,033	58.1	399	5.8	
Estrogen plus progestin only	52	2.9	1,561	22.6	
Multiple/other formulations	129	7.3	336	4.9	
Stage, %					
Local	1,183	66.5	4,421	64.0	0.04
Regional	423	23.8	1,797	26.0	
Distant	23	1.3	140	2.0	
Unknown	149	8.4	555	8.0	
Histology, %					
Ductal	1,407	79.1	5,429	78.5	0.01
Lobular/non-ductal	122	6.9	615	8.9	
Other	244	13.7	847	12.3	

^a^TAHBSO, total abdominal hysterectomy and bilateral salpingo-oophorectomy; ^b^*P* value for *t* tests of continuous variables and chi-square tests of categorical variables; ^c^among parous women only (N = 1,541 (86.7%) who reported TAHBSO; N = 6,016 (87.0%) with intact uterus and ovaries).

### All-cause mortality

**Figure 1:**
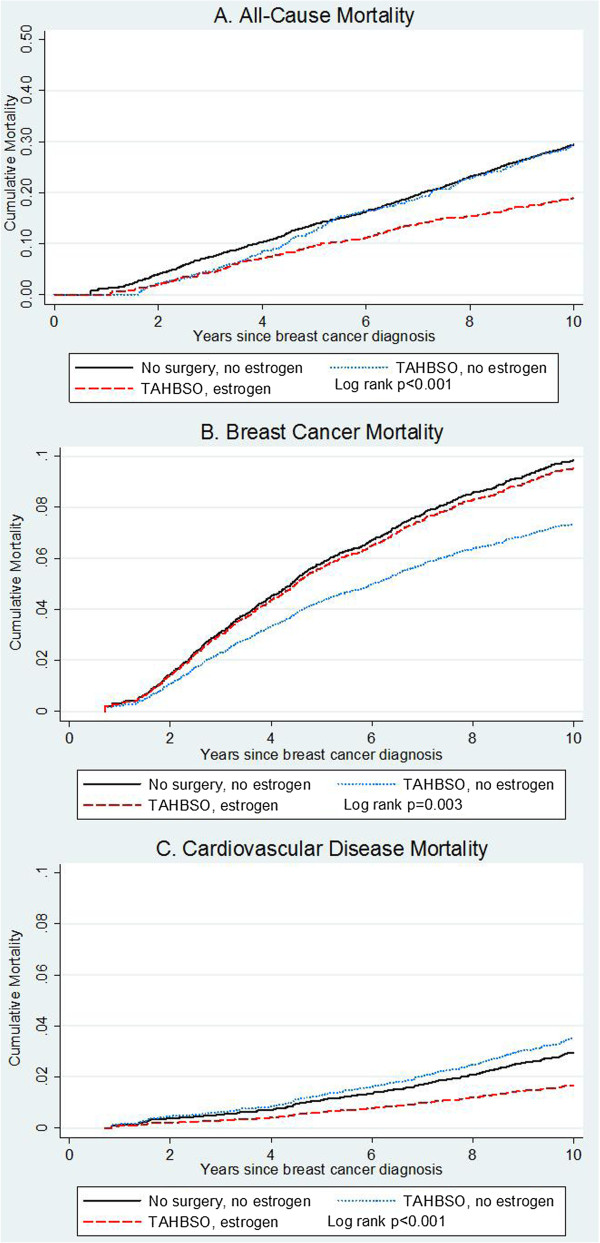
**Cumulative mortality due to (A) all causes, (B) breast cancer, and (C) cardiovascular disease.** Mortality curves are shown according to pre-diagnosis surgery status: intact uterus and ovaries (‘no surgery’) versus total abdominal hysterectomy with bilateral oophorectomy (‘TAHBSO’), and unopposed estrogen use (never/ever).

TAHBSO was associated with a 16% decrease in mortality from all causes (HR = 0.84; 95% confidence interval (CI): 0.76, 0.92). All-cause mortality did not appear to vary according to age at surgery. The inverse association between pre-diagnosis TAHBSO and all-cause mortality was of greater magnitude among women who reported using estrogen therapy (HR = 0.78; 95% CI: 0.69, 0.89) compared to no estrogen use (HR = 0.92; 95% CI: 0.80, 1.05) (Table 2, Figure 1A). All-cause mortality risk estimates were similar across categories of estrogen duration (HR = 0.81; 95% CI: 0.65, 1.02 for 0.5 to 4.9 years; HR = 0.75; 95% CI: 0.56, 0.99 for 5 to 9.9 years; and HR = 0.77; 95% CI: 0.65, 0.92 for ≥10 years) (Table 2).

**Table 2:** Hazard ratios (95% CI) for mortality according to gynecologic surgery and estrogen use prior to breast cancer diagnosis, Collaborative Breast Cancer Studies

	**Person-years**	***All-cause mortality***		***Breast cancer mortality***		***Cardiovascular disease mortality***	
		**Deaths**	**HR (95% CI)**^**a**^	**Deaths**	**HR (95% CI)**^**b**^	**Deaths**	**HR (95% CI)**^**b**^
Intact uterus and ovaries, never hormone use	43,221	1,877	1	661	1	326	1
TAHBSO^c^	17,549	557	0.84 (0.76, 0.92)	198	0.76 (0.64, 0.90)	94	0.84 (0.67, 1.06)
*Age at surgery:*							
≤ 45 years	6,459	218	0.86 (0.75, 0.99)	71	0.85 (0.66, 1.09)	43	1.04 (0.75, 1.44)
> 45 years	11,090	339	0.82 (0.73, 0.92)	127	0.86 (0.71, 1.05)	51	0.73 (0.54, 0.98)
*Unopposed estrogen use:*							
Never used hormones	5,397	241	0.92 (0.80, 1.05)	59	0.73 (0.55, 0.96)	58	1.19 (0.89, 1.58)
Used unopposed estrogen	10,245	270	0.78 (0.69, 0.89)	120	0.97 (0.80, 1.18)	31	0.57 (0.39, 0.83)
*Duration of estrogen use:*							
6 months- 4.9 years	2,765	80	0.81 (0.65, 1.02)	39	0.73 (0.56, 0.96)	12	0.72 (0.41, 1.29)
5 - 9.9 years	2,349	49	0.75 (0.56, 0.99)	23	1.19 (0.84, 1.66)	6	0.68 (0.30, 1.55)
≥ 10 years	5,131	141	0.77 (0.65, 0.92)	58	0.76 (0.50, 1.19)	13	0.45 (0.26, 0.78)

^a^Hazard ratios adjusted for age, study enrollment years, state, body mass index, cigarette smoking, family history of breast cancer, and stage at diagnosis; ^b^cumulative incidence subhazard ratios adjusted for age, study enrollment years, state, body mass index, cigarette smoking, family history of breast cancer, and stage at diagnosis; ^c^TAHBSO, total abdominal hysterectomy and bilateral salpingo-oophorectomy.

All-cause mortality among women who underwent pre-diagnosis TAHBSO and used estrogen therapy appeared to vary with follow-up time (Figure 1A): during the first four years after breast cancer diagnosis, estrogen use did not seem to influence mortality, whereas approximately five years after breast cancer diagnosis estrogen therapy appeared to confer a survival advantage. Therefore, we examined five-year mortality risk separately from deaths that occurred less than five years after diagnosis. TAHBSO alone was not associated with decreased all-cause mortality within the first five years after diagnosis (HR = 0.93; 95% CI: 0.78, 1.10). However, among five-year breast cancer survivors, we observed a 20% decrease in mortality risk among women who reported pre-diagnosis TAHBSO (HR = 0.80; 95% CI: 0.72, 0.90), particularly among women who also used estrogen therapy (HR = 0.72; 95% CI: 0.62, 0.85) (Table 3).

**Table 3:** Hazard ratios (95% CI) for mortality according to age at diagnosis and follow-up period, Collaborative Breast Cancer Studies

	**Person-years**	***All-cause mortality***		***Breast cancer mortality***		***Cardiovascular disease mortality***	
		**Deaths**	**HR (95% CI)**^**a**^	**Deaths**	**HR (95% CI)**^**b**^	**Deaths**	**HR (95% CI)**^**b**^
***Follow-up period:***							
***5-year mortality***							
Intact uterus and ovaries, never hormone use	15,462	537	1	331	1	59	1
TAHBSO^c^	6,165	170	0.93 (0.78, 1.10)	103	0.93 (0.74, 1.16)	19	0.94 (0.55, 1.58)
TAHBSO, Never used hormones	1,857	64	0.91 (0.70, 1.19)	31	0.79 (0.54, 1.16)	10	1.14 (0.58, 2.24)
TAHBSO, Used unopposed estrogens	3,596	90	0.93 (0.75, 1.17)	61	1.03 (0.77, 1.36)	7	0.70 (0.32, 1.55)
***Mortality among 5-year survivors***							
Intact uterus and ovaries, never hormone use	27,759	1,340	1	330	1	267	1
TAHBSO^c^	11,384	387	0.80 (0.72, 0.90)	95	0.81 (0.64, 1.02)	75	0.82 (0.63, 1.06)
TAHBSO, Never used hormones	3,541	177	0.82 (0.78, 1.07)	28	0.69 (0.46, 1.01)	48	1.19 (0.87, 1.63)
TAHBSO, Used unopposed estrogens	6,649	180	0.72 (0.62, 0.85)	59	0.92 (0.69, 1.23)	24	0.53 (0.35, 0.81)
***Age at diagnosis:***							
***50-64 years***^***d***^							
Intact uterus and ovaries, never hormone use	20,968	593	1	326	1	60	1
TAHBSO^c^	9,559	189	0.78 (0.66, 0.91)	103	0.78 (0.62, 0.98)	15	0.61 (0.34, 1.09)
TAHBSO, Never used hormones	1,890	47	0.80 (0.59, 1.08)	22	0.68 (0.43, 1.07)	6	0.97 (0.42, 2.24)
TAHBSO, Used unopposed estrogens	6,391	115	0.75 (0.61, 0.92)	68	0.85 (0.65, 1.12)	5	0.32 (0.13, 0.80)
***≥ 65 years***							
Intact uterus and ovaries, never hormone use	22,253	1,284	1	335	1	296	1
TAHBSO^c^	7,990	368	0.86 (0.77, 0.97)	95	0.95 (0.75, 1.20)	79	0.90 (0.69, 1.15)
TAHBSO, Never used hormones	3,507	194	0.95 (0.82, 1.11)	37	0.76 (0.54, 1.08)	52	1.21 (0.89, 1.64)
TAHBSO, Used unopposed estrogens	3,854	155	0.80 (0.67, 0.94)	52	1.15 (0.85, 1.54)	26	0.65 (0.43, 0.98)

^a^Hazard ratios adjusted for age, study enrollment years, state, body mass index, cigarette smoking, family history of breast cancer, and stage at diagnosis; ^b^cumulative incidence subhazard ratios adjusted for age, study enrollment years, state, body mass index, cigarette smoking, family history of breast cancer, and stage at diagnosis; ^c^TAHBSO, total abdominal hysterectomy with bilateral salpingo-oophorectomy; ^d^due to the low number of cardiovascular disease mortality events among women ages 50 to 64 at diagnosis, full multivariate adjustment was not performed. CVD mortality cumulative incidence subhazard ratios are adjusted for age, study enrollment years, and stage at diagnosis.

Mortality due to causes other than breast cancer may be more common among women diagnosed at ages 65 and older [4,27]. With this consideration, we also evaluated HRs for cause-specific mortality according to age at diagnosis. Overall, TAHBSO was associated with decreased all-cause mortality among both women who were diagnosed at ages 50 to 64 (HR = 0.78; 95% CI: 0.66, 0.91) and ages ≥65 (HR = 0.86; 95% CI: 0.77, 0.97). When evaluated according to estrogen use, estimates remained statistically significant only among estrogen users (HR = 0.75; 95% CI: 0.61, 0.92 among women ages 50 to 64 and HR = 0.80; 95% CI: 0.67, 0.94 among women ≥65) (Table 3).

### Breast cancer-specific mortality

In multivariate competing-risk regression models, pre-diagnosis TAHBSO was associated with a 24% reduction in breast cancer mortality (HR = 0.76; 95% CI: 0.64, 0.90). No variation was observed according to age at surgery. When evaluated according to estrogen use, decreased breast cancer mortality risk was observed among women who reported TAHBSO and never used hormones (HR = 0.73; 95% CI: 0.55, 0.96) but not among women who used unopposed estrogens (HR = 0.97; 95% CI: 0.80, 1.18) (Table 2, Figure 1B). We did not observe a consistent pattern of breast cancer-specific mortality risk according to duration of estrogen use (Table 2).

When evaluated according to time since diagnosis, TAHBSO was associated with a suggested decrease in breast cancer mortality among five-year survivors (HR = 0.81; 95% CI: 0.64, 1.02 for TAHBSO overall; HR = 0.69; 95% CI: 0.46, 1.01 for women who reported TAHBSO and never hormone use). In analyses stratified according to age at diagnosis, TAHBSO was associated with statistically significant reductions in breast cancer mortality only among women ages 50 to 64 at diagnosis (HR = 0.78; 95% CI: 0.62, 0.98) (Table 3).

### Cardiovascular disease-specific mortality

Overall, the association between TAHBSO and risk of death due to CVD was not statistically significant (HR = 0.84, 0.67, 1.06) in multivariate competing-risk regression models. When evaluated according to age at surgery, TAHBSO at ages ≤45 years was not related to CVD mortality (HR = 1.04; 95% CI: 0.75, 1.44), but decreased CVD mortality was observed among women who reported TAHBSO at older ages (HR = 0.73; 95% CI: 0.54, 0.98). Women who reported TAHBSO and estrogen therapy use had a 43% decrease in CVD mortality (HR = 0.57; 95% CI: 0.39, 0.83); and those who reported at least 10 years of estrogen use had an estimated 55% reduction in CVD mortality (HR = 0.45; 95% CI: 0.26, 0.78). Conversely, women who reported TAHBSO and did not use estrogen had a non-statistically significant 19% increase in CVD mortality risk (95% CI: 0.89, 1.58) (Table 2, Figure 1C).

Fewer CVD deaths occurred within the first five years of diagnosis or among women who were 50 to 64 years at breast cancer diagnosis compared to five-year survivors and older women. Among five-year breast cancer survivors, those who reported TAHBSO and unopposed estrogen use had an estimated 47% reduction in CVD mortality (HR = 0.53; 95% CI: 0.35, 0.81). Sample sizes were insufficient to calculate fully adjusted HRs for CVD mortality among women ages 50 to 64 at diagnosis, estimates in this group are adjusted for study enrollment years, age and stage at diagnosis only. A 68% decrease in CVD mortality was estimated among women ages 50 to 64 at diagnosis who reported previous TAHBSO and estrogen use (HR = 0.32; 95% CI: 0.13, 0.80). Among women who were ≥65 at diagnosis, we observed a 35% decrease in CVD mortality associated with TAHBSO and estrogen use (HR = 0.65; 95% CI: 0.43, 0.98) (Table 3).

In sensitivity analyses restricted to participants residing in Wisconsin (N = 5,992) or those with local disease (N = 5,604), our findings were essentially unchanged compared to the overall analysis.

## Discussion

In this study, postmenopausal women who had a hysterectomy with bilateral oophorectomy prior to breast cancer diagnosis had an estimated 16% reduction in all-cause mortality and 24% decrease in breast cancer mortality compared to women who had an intact uterus and never used hormones, irrespective of age at surgery. Cause-specific mortality associations differed according to post-surgical estrogen use. Breast cancer mortality was lowest among those who did not use estrogens after surgery, while cardiovascular disease and all-cause mortality were lowest among estrogen users. These patterns were more apparent among five-year breast cancer survivors compared to women who were within five years of a breast cancer diagnosis. These findings indicate that prior oophorectomy and estrogen use can impact cause of death among breast cancer survivors.

Results from historical [9] and recent [10,12] studies examining the association between pre-diagnosis bilateral oophorectomy and subsequent mortality in the general population have been mixed. In an early study using data from the Connecticut Cancer Registry, bilateral oophorectomy was associated with improved five-year survival after breast cancer diagnosis (72% versus 58%) [9]. However, in a recent analysis of data from the Nurses’ Health Study, no association was reported for bilateral oophorectomy and hysterectomy and breast cancer mortality (HR = 0.94; 95% CI: 0.70 to 1.26) based on 230 breast cancer deaths during a 24-year follow-up. Age-adjusted hazard ratios for breast cancer mortality ranged from 0.78 for women less than 45 years at oophorectomy to 1.16 for women age 45 to 54 years at surgery [10]. The start of follow-up was defined as the age at surgery for oophorectomized women [10]; mortality hazard ratios therefore incorporate the reduction in breast cancer incidence associated with premenopausal oophorectomy, which could reduce the death rate by lowering the number of breast cancers that develop initially. In our analysis, follow-up started at breast cancer diagnosis; therefore, our observed associations between TAHBSO and mortality are not influenced by the decreased risk of developing breast cancer associated with TAHBSO and demonstrate survival benefits after breast cancer has already been diagnosed.

Previous studies have suggested that use of estrogen therapy after TAHBSO can mitigate the potential increased risk of CVD associated with early surgical menopause [12,14,16]. We hypothesized that women who reported TAHBSO without estrogen use would have an elevated risk of cardiovascular mortality relative to the comparison group of women with an intact uterus and ovaries who never used hormones. The strong inverse association we observed between estrogen use after TAHBSO and cardiovascular mortality among breast cancer survivors in our study population was unexpected. Clinical trial results have refuted a cardioprotective effect of estrogen therapy in healthy women overall, although they have not often focused specifically on those with a prior TAHBSO [28]. One explanation of our results could be that women who are susceptible to developing severe CVD after TAHBSO with estrogen therapy do so prior to developing breast cancer, which would result in the appearance of a protective effect of estrogens.

Limitations to this analysis include potential misclassification of exposure based on self-reported information. However, our confidence in these data is strengthened by the high reliability of the study questionnaire. In a sequential sample of 195 women without breast cancer who were re-interviewed after an average of 3.5 months, hysterectomy and oophorectomy status were highly reproducible (κ = 0.98; 95% CI: 0.95, 1.00) [29]. Although validation data were not available here, agreement between medical reports and self-reported bilateral oophorectomy status has ranged from 70 to 96% in other studies [30-33]. In a subset of these data [22] and elsewhere [34-39], self-reported postmenopausal hormone use has also been shown to be a reliable and valid measure for use in epidemiological studies.

In the Women’s Contraceptive and Reproductive Experiences case-control study, bilateral oophorectomy was associated with stronger reductions in the incidence of estrogen receptor (ER)+/progesterone receptor (PR)+ breast cancers (OR = 0.55; 95% CI: 0.45, 0.68) compared to ER-/PR- tumors (OR = 0.82; 95% CI: 0.63, 1.07) [40]. In our study, information on hormone receptor status and treatment regimen was not available; however, the age and postmenopausal status of participants at breast cancer diagnosis suggest that the majority were likely to be hormone receptor positive and to have received endocrine therapy [41]. If the inverse association between TAHBSO and mortality were limited to ER+/PR+ tumors, the inclusion of other tumor types in our analyses could have attenuated our results. Our confidence in these results is strengthened by sensitivity analyses demonstrating equivalent associations for local disease only and all breast cancer diagnoses (adjusted for stage).

Our exposure assessment was based on a single interview conducted approximately one year after breast cancer diagnosis. Some study participants may have undergone TAHBSO after this time; these surgeries would have been performed after menopause and would likely exert a lesser effect on mortality risk, although intact ovaries continue to produce small amounts of androgens after menopause and could influence chronic disease risk [42,43]. The lack of information on post-diagnosis estrogen therapy is unlikely to be a major issue as it is generally not recommended for women with a breast cancer diagnosis [44]. Our analyses were specific to women with a breast cancer diagnosis at ages 50 and older, and may not be generalizable to women who have not had breast cancer, who were diagnosed at younger ages, or who underwent bilateral oophorectomy after diagnosis.

## Conclusions

This analysis provides evidence of a beneficial effect of pre-diagnosis TAHBSO on overall mortality in a large, population-based cohort of breast cancer survivors. Given that an estimated 15% of US women in the general population undergo elective bilateral oophorectomy by age 60 [19], and breast cancer occurs in one in eight women during their lifetime [1], this is not an uncommon scenario. These findings illustrate the need to monitor both cancer and non-cancer outcomes in breast cancer survivors.

## Abbreviations

BMI: Body mass index; CI: Confidence interval; CVD: Cardiovascular disease; ER: Estrogen receptor; HR: Hazard ratio; PR: Progesterone receptor; SD: Standard deviation; TAHBSO: Total abdominal hysterectomy bilateral salpingo-oophorectomy.

## Competing interests

The authors declare that they have no competing interests.

## Authors’ contributions

This analysis was conceived and proposed by HN and KV. HN, ATD, PN, KE, LT, JH, and KV contributed substantially to the design and interpretation of data. Data analysis was performed by HN and JH. HN, ATD, PN, KE, LT, JH, and KV were involved in revising the manuscript for intellectual content and gave final approval for publication.
